# VEGFR3 is required for button junction formation in lymphatic vessels

**DOI:** 10.1016/j.celrep.2023.112777

**Published:** 2023-07-16

**Authors:** Melanie Jannaway, Drishya Iyer, Diandra M. Mastrogiacomo, Kunyu Li, Derek C. Sung, Ying Yang, Mark L. Kahn, Joshua P. Scallan

**Affiliations:** 1 Department of Molecular Pharmacology and Physiology, Morsani College of Medicine, University of South Florida, Tampa, FL 33612, USA; 2 Cardiovascular Institute, Department of Medicine, Perelman School of Medicine, University of Pennsylvania, Philadelphia, PA 19104, USA; 3 Lead contact

## Abstract

Lymphatic capillaries develop discontinuous cell-cell junctions that permit the absorption of large macromolecules, chylomicrons, and fluid from the interstitium. While excessive vascular endothelial growth factor 2 (VEGFR2) signaling can remodel and seal these junctions, whether and how VEGFR3 can alter lymphatic junctions remains incompletely understood. Here, we use lymphatic-specific *Flt4* knockout mice to investigate VEGFR3 signaling in lymphatic junctions. We show that loss of *Flt4* prevents specialized button junction formation in multiple tissues and impairs interstitial absorption. Knockdown of *FLT4* in human lymphatic endothelial cells results in impaired NOTCH1 expression and activation, and overexpression of the NOTCH1 intracellular domain in *Flt4* knockout vessels rescues the formation of button junctions and absorption of interstitial molecules. Together, our data reveal a requirement for VEGFR3 and NOTCH1 signaling in the development of button junctions during postnatal development and may hold clinical relevance to lymphatic diseases with impaired VEGFR3 signaling.

## In brief

Lymphatic capillaries form discontinuous junctions called buttons to enable absorption of interstitial contents. Jannaway et al. show that vascular endothelial growth factor receptor 3 (VEGFR3) expression is required for the formation of button junctions and that NOTCH1 signaling can fully rescue button formation in the absence of VEGFR3.

## Graphical abstract

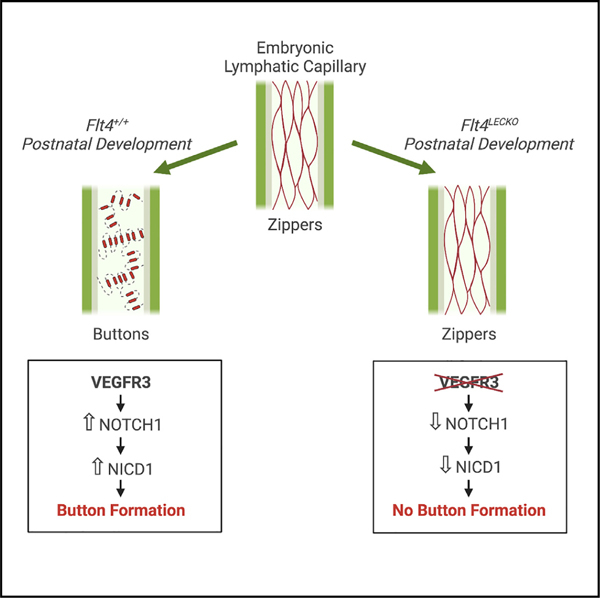

## INTRODUCTION

The lymphatic vasculature absorbs fluid, protein, and cells from the interstitium and transports it back into the blood circulation. However, inherited genetic mutations can impair lymphatic vessel function and lymph flow to cause a collection of diseases known as congenital lymphedema. While several gene mutations have been identified in the past decade in congenital lymphedema patients,^1^ with many of these occurring in signaling pathways that regulate the development of lymphatic valves,^2^ there are no pharmacological therapies for these patients.

To prevent or clear edema, lymphatic vessels absorb the majority of interstitial fluid. Interstitial fluid absorption by lymphatic capillaries is permitted via the remodeling of continuous junction proteins, reminiscent of zippers, into punctate discontinuous junctions that have been referred to as buttons.^3^ Button junctions first begin to form at E17.5 in the skin lymphatic vasculature and mature by 3 weeks after birth.^4^ While angiopoietin-2 and DLL4 signaling in lymphatic endothelial cells (LECs) regulate button junctions in lymphatic capillaries in the skin and intestine, respectively,^5,6^ the molecular mechanisms required for button formation during development are still poorly understood. Recently, genetic deletion of two VEGFA receptors, *Flt1* and *Nrp1,* from vascular endothelium resulted in the zippering of existing intestinal lacteal buttons and prevented high-fat-diet-induced obesity in mice.^7^ The proposed cause of this zippering was increased VEGFA ligand availability and thus excessive activation of VEGFR2. This role for VEGFR2 was confirmed by a new model of viral infection that caused button junctions to revert to continuous zipper-like junctions.^8^ In contrast, a clear mechanism for VEGFR3 in button formation has not been elucidated.

Here, we identify VEGFR3 as a major regulator of button junction development during postnatal life. To better understand the role of VEGFR3 in lymphatic function, we induced the deletion of *Flt4* from all LECs postnatally (*Flt4*^*iLECKO*^). Our data reveal that VEGFR3 is required for button formation during a critical period of postnatal development but is later dispensable for button junction maintenance. We also show that overexpression of the NOTCH1 intracellular domain (NICD) is sufficient to rescue button formation in the absence of VEGFR3 and restores the function of lymphatic capillaries to absorb interstitial fluid. Our findings reveal that a VEGFR3/NOTCH1 signaling axis is required for button formation and interstitial absorption. Furthermore, the most common form of congenital lymphedema, Milroy disease, is caused by dominant-negative point mutations in the VEGFR3 gene, and these patients exhibit dysfunctional interstitial absorption. Thus, our findings here hold potential clinical relevance to this disease.

## RESULTS

### Deletion of *Flt4* prevents button formation in the diaphragm and ear skin

**Figure 1.**
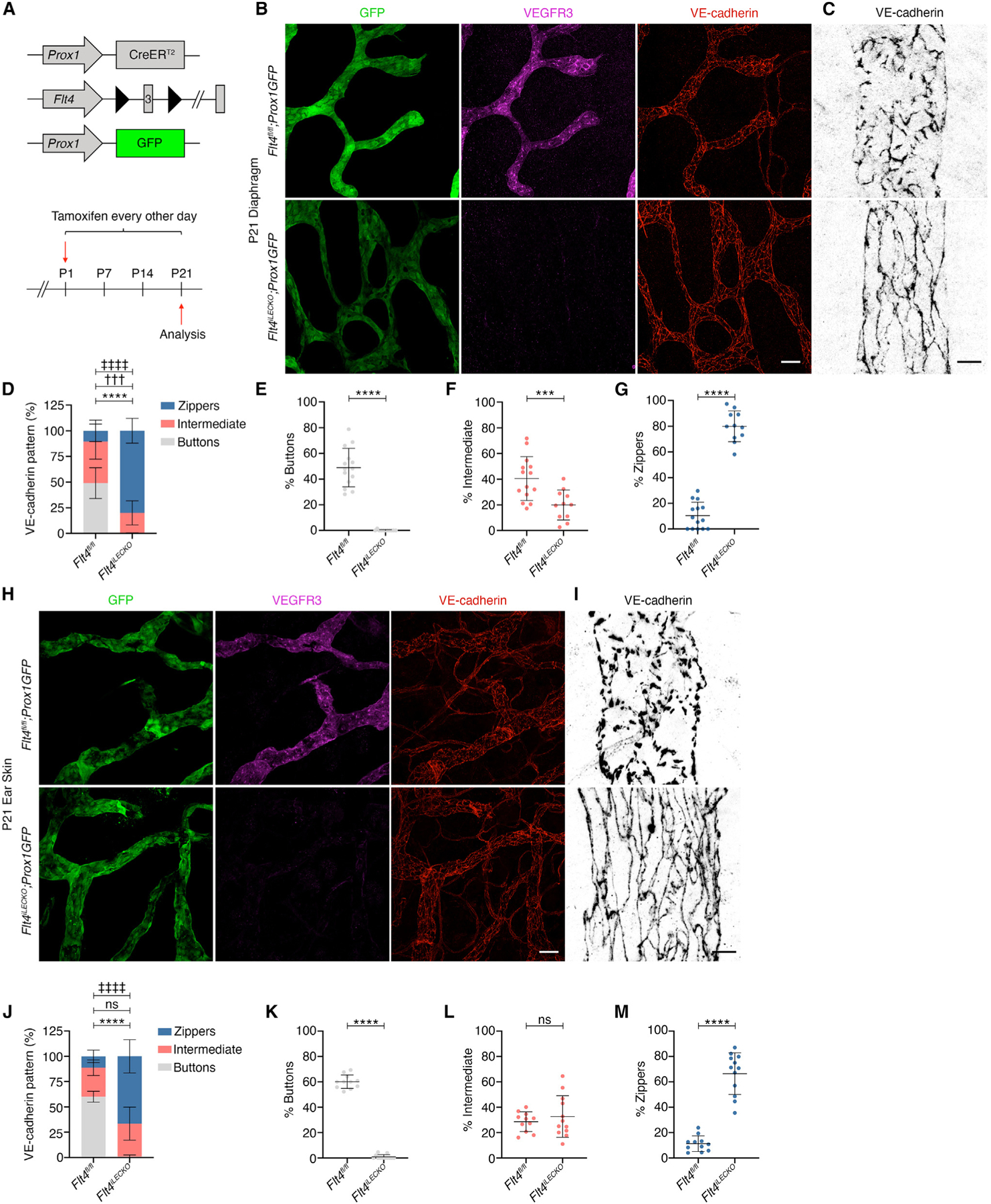
Lymphatic-specific deletion of *Flt4* prevents the appearance of buttons in the diaphragm and ear skin (A) Tamoxifen injection procedure for deletion of *Flt4* to assess button formation. (B) P21 diaphragms immunostained for GFP (green), VEGFR3 (magenta), and VE-cadherin (red). (C) Higher magnification images of lymphatic vessel junction morphology. (D) Quantification of button, intermediate, and zipper junctions in *Flt4*^*fl/fl*^ (N = 7 mice; n = 11 fields of view [FOVs]) and *Flt4*^*iLECKO*^ (N = 6 mice; n = 12 FOVs) mice. (E–G) A breakdown of the graph in (D) for the indicated junction types. (H) P21 ear skin was stained for GFP (green), VEGFR3 (magenta), and VE-cadherin (red). (I) Higher magnification of VE-cadherin at intercellular junctions. (J) Junction morphology quantification in *Flt4*^*fl/fl*^ (N = 5 mice; n = 11 FOVs) and *Flt4*^*iLECKO*^ (N = 6; n = 12 FOVs) mice. (K–M) Individual graphs for each of the three junction types presented in (J). Two-way ANOVA with Sidak’s test. ****p < 0.0001 for buttons; †††p < 0.001 for intermediate; ‡‡‡‡p < 0.0001 for zippers; ns, non-significant for intermediate. All data are presented as mean ± SD. Scale bar in (B) and (H) represents 50 μm and in (C) and (I) represents 10 μm. FOVs, fields of view.

*Flt4* was deleted from LECs by crossing *Flt4*^*fl/fl* 9^ with a lymphatic-specific *Prox1CreER*^*T2*^ strain.^10^ While this Cre strain can delete genes highly efficiently with two injections of tamoxifen (TM),^11,12^ we and others have found that inactivation of *Flt4* led to the reappearance of VEGFR3-competent LECs.^13^ Therefore, we administered TM every other day from P1 until analysis at P21 (Figure 1A) to *Flt4*^*fl/fl*^ control and *Prox1CreER*^*T2*^*; Flt4*^*fl/fl*^ knockout littermates (hereafter: *Flt4*^*iLECKO*^). To assess button junction development, we located tissues rich in lymphatic capillaries that remodeled their junctions into buttons. The lymphatic vessels of the diaphragm were previously used as a model for postnatal button formation.^5^ All lymphatic vessels in the diaphragm of control and *Flt4*^*iLECKO*^ pups expressed the lymphatic capillary marker LYVE1 (Figure S1) and the Prox1-GFP reporter,^14^ which was used to visualize lymphatic vessel morphology (Figure 1B). Similar to a previous report, postnatal deletion of *Flt4* led to lymphatic vessel hyperplasia.^13^ Confirming successful deletion of *Flt4*, we found that VEGFR3 was absent from all lymphatic vessels in the P21 *Flt4*^*iLECKO*^ diaphragms (Figure 1B). Immunostaining for the endothelial-specific adherens junction protein VE-cadherin is commonly used to identify button junctions.^3^ By P21, mature button junctions demarcated by discontinuous VE-cadherin staining were found in control mice (Figure 1C), whereas continuous VE-cadherin staining revealed the presence of zippers in the lymphatic vessels in the *Flt4*^*iLECKO*^ diaphragm (Figure 1C). We quantified button development by measuring the junction lengths and orientation to classify them as buttons, zippers, or intermediate junctions (Figure 1C). Approximately 49% of the total junctions are buttons in control lymphatic vessels compared to <1% in the *Flt4*^*iLECKO*^ lymphatic vessels. In contrast, zipper junctions accounted for 10% in control vessels but increased to 80% in the *Flt4*^*iLECKO*^ vessels (Figures 1D–1G). Together, these data indicate that VEGFR3 is required for button formation in the diaphragm.

To confirm a requirement for VEGFR3 in button formation, we examined button junctions in the ear skin at P21 (Figures 1H and S2). Because the mouse ear develops entirely postnatally, this enabled the deletion of *Flt4* before lymphatic capillaries form. Staining for VEGFR3 confirmed its deletion from lymphatics in the *Flt4*^*iLECKO*^ ear skin (Figure 1H). As in the diaphragm, ear skin lymphatic capillaries possess mature VE-cadherin^+^ button junctions in P21 controls, but buttons completely failed to form in the *Flt4*^*iLECKO*^ ears (Figure 1I). When junction types were quantified, the *Flt4*^*iLECKO*^ mice had significantly more zippers (66% vs. 12%) and fewer buttons (<1% vs. 60%) compared to littermate controls, indicating that VEGFR3 is required for button formation in the ear skin (Figures 1J–1M). Because button junctions have been investigated in the intestinal lacteals recently and to examine a third tissue type, we found that the lacteals of *Flt4*^*iLECKO*^ mice lacked button junctions and were zippered (Figure S3).

### VEGFR3 is not required to maintain button junctions throughout life

**Figure 2.**
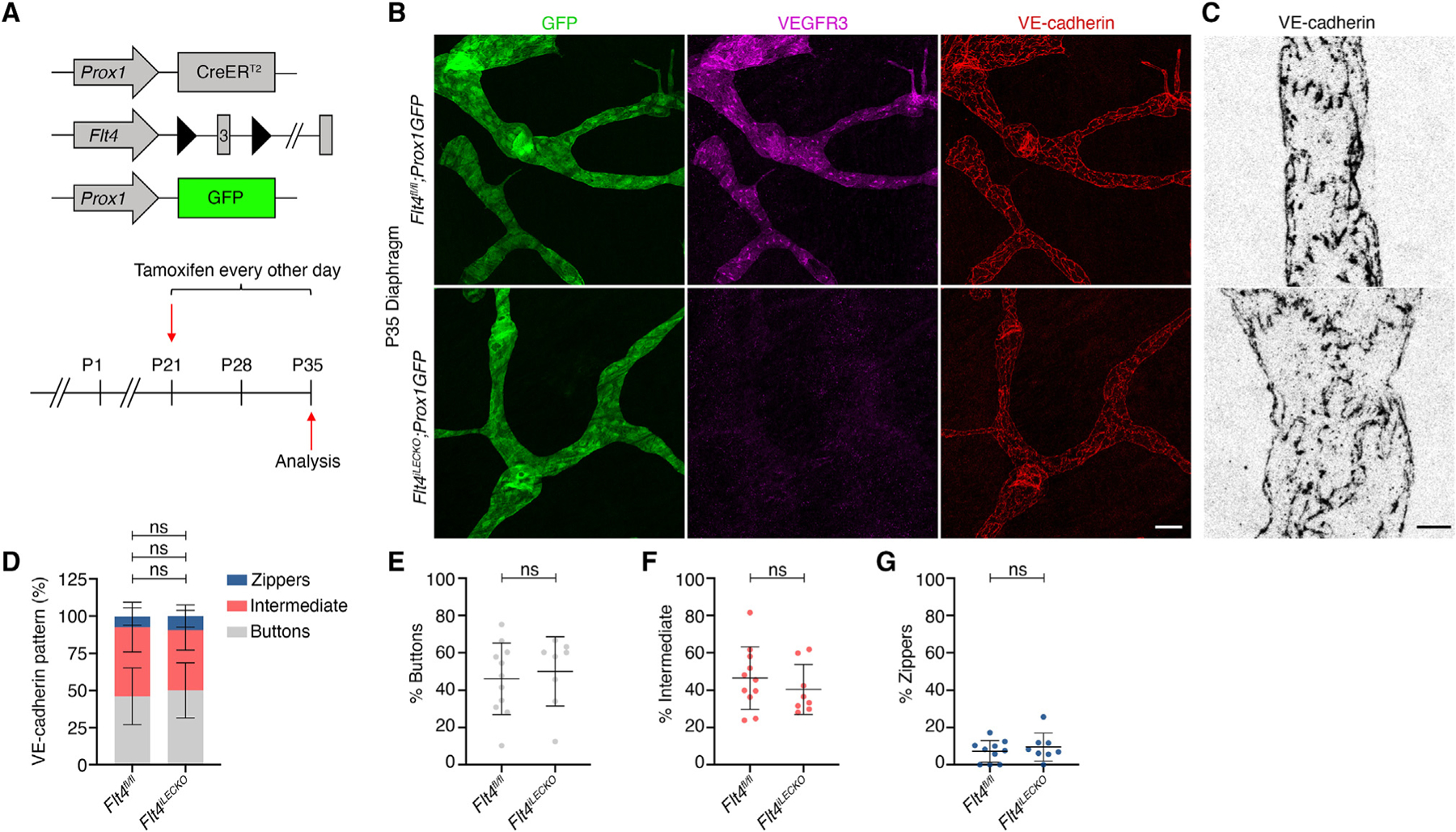
VEGFR3 is not required for button maintenance (A) Tamoxifen injection procedure to delete *Flt4* after buttons have formed to investigate button maintenance. (B) P35 diaphragms were immunostained for GFP (green), VEGFR3 (magenta), and VE-cadherin (red). (C) Higher magnification of junction phenotype in lymphatic capillaries. (D) Quantification of junction phenotype in *Flt4*^*fl/fl*^ (N = 5 mice; n = 11 FOVs) and *Flt4*^*iLECKO*^ (N = 5 mice; n = 8 FOVs) mice. (E–G) Individual graphs of each junction type presented in (D). Two-way ANOVA with Sidak’s test. ns, non-significant. All data are presented as mean ± SD. Scale bar in (B) represents 50 μm, and in (C), it represents 10 μm. FOVs, fields of view.

Whether button junctions are stable throughout life or require constant cell signals to be maintained, similar to lymphatic valves,^11,15^ is unknown. Infection of adult mice with *M. pulmonis* or *vaccinia* virus can transform buttons back into zippers in tracheal lymphatics and skin lymphatics, respectively, suggesting that the button junction phenotype is not permanent.^4,8^ To test whether VEGFR3 signaling maintains button junctions, we induced *Flt4* deletion at P21 and analyzed junction types in diaphragm lymphatic capillaries 2 weeks later at P35 (Figure 2A). We confirmed efficient deletion of VEGFR3 at P35 in *Flt4*^*iLECKO*^ mice (Figure 2B). VE-cadherin staining revealed no significant difference in button, intermediate, or zipper junctions between controls and knockouts (Figures 2B–2G), indicating that VEGFR3 signaling is not required to maintain button junctions after their formation.

### Loss of VEGFR3 inhibits NOTCH signaling

**Figure 3.**
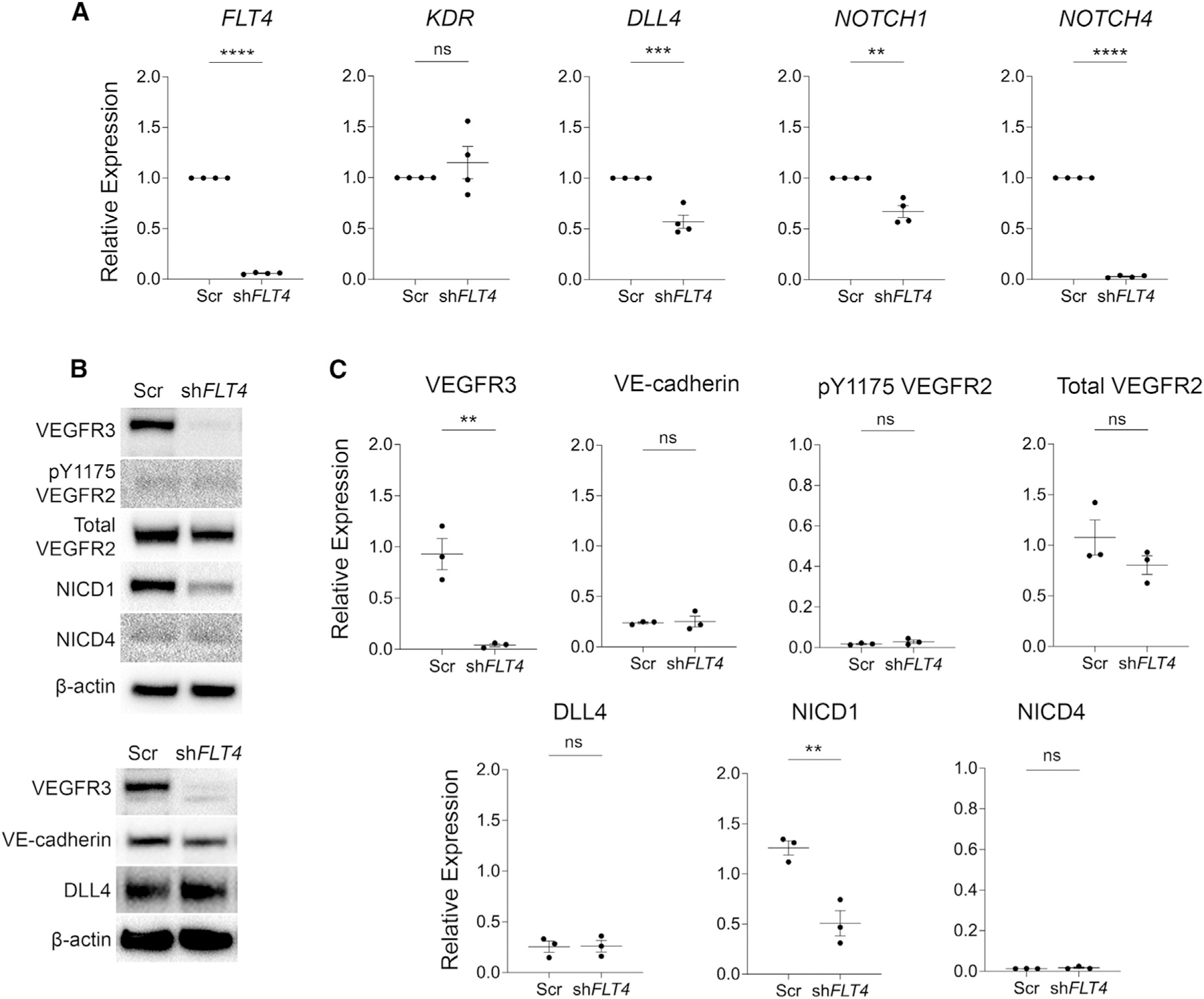
*FLT4* knockdown inhibits Notch signaling and expression (A) qRT-PCR analysis for members of the Notch signaling pathway and other putative signaling pathways associated with VEGFR3 in cultured human dermal lymphatic endothelial cells. (B) Western blot for VEGFR3, pVEGFR2, VEGFR2, cleaved NOTCH1, cleaved NOTCH4, VE-cadherin, and DLL4 to assess activation of these signaling pathways. (C) Quantification of the western blot data in (B). **p < 0.01; ***p < 0.001; ****p < 0.0001; ns, non-significant. Student’s unpaired t test. All data are presented as mean ± SEM. All experiments were performed n = 3–4 times.

To investigate the downstream mechanisms by which VEGFR3 regulates button junction formation, we performed lentiviral knockdown of *FLT4* (Figure 3A) in human dermal lymphatic endothelial cells (hdLECs) and probed genes of interest with qRT-PCR. Although VEGFR2 activation has been shown to zipper the button junctions,^7,8^ we found that *KDR* expression did not change (Figure 3A).

VEGFR3 has been closely linked with Notch signaling in the vasculature.^13,16^ VEGFR3 induces Delta-like ligand 4 (DLL4) expression in both the blood^17^ and lymphatic^13^ vasculature. In blood and lymphatic endothelium, DLL4 is the ligand for NOTCH receptor 1 and 4.^16,18,19^ We found that the expression of *DLL4*, *NOTCH1*, and *NOTCH4* (Figure 3A) were significantly decreased in *FLT4* knockdown hdLECs.

To confirm these findings at the protein level, western blot was performed and quantified (Figures 3B and 3C). Neither total VEGFR2 nor phosphorylated VEGFR2 were altered with *FLT4* knockdown. VE-cadherin protein levels were not significantly changed. Upon ligand binding, the Notch intracellular domain (NICD) is cleaved and translocates to the nucleus to initiate gene transcription.^20^ Western blot for cleaved NICD1 and NICD4 in sh*FLT4*-treated cells revealed that NICD1 activation was significantly decreased, whereas NICD4 had very low basal expression that did not change (Figures 3B and 3C). In contrast to the RT-PCR data, there was no significant change in DLL4 at the protein level. Our results indicate that NOTCH1 is a major target of VEGFR3 signaling in LECs. Further, our data imply that DLL4 may not be the ligand to initiate button formation or that NOTCH ligands are tissue specific.

### Button formation defect is not induced by VEGFR2 activity

Although our *in vitro* data showed no difference in VEGFR2 expression or activation, VEGFR ligands *in vivo* could potentially be elevated leading to VEGFR2 hyperactivation. To rule this out, we analyzed the junctions in *Prox1CreER*^*T2*^*;Kdr*^*fl/fl*^ mice (hereafter: *Kdr*^*iLECKO*^). *Kdr* was deleted from P1 with the same dose and frequency of TM as before (Figure S4A). Staining for VEGFR2 confirmed its deletion from LECs at P21 (Figure S4B). VE-cadherin staining revealed no difference in button junctions between the *Kdr*^*fl/fl*^ controls and *Kdr*^*iLECKO*^ (Figures S4B and S4C). Quantification of junction types confirmed that *Kdr* knockouts did not have any significant changes in the number of button (Figures S4D and S4E), intermediate (Figures S4D–S4F), or zipper junctions (Figures S4D–S4G).

Next, we generated *Prox1CreER*^*T2*^*;Kdr*^*fl/fl*^*;Flt4*^*fl/fl*^ mice (hereafter: *Flt4*^*iLECKO*^*;Kdr*^*iLECKO*^), simultaneously deleting both receptors from P1 (Figure S5A). In support of our *in vitro* data, the PROX1^+^ lymphatic capillaries lacking VEGFR2 and VEGFR3 were still unable to form button junctions (Figures S5B–S5D), mirroring the *Flt4* single knockouts. Quantification showed significantly fewer buttons (~51% to ~3%) (Figure S5E), significantly fewer intermediate junctions (Figure S5F; 36%–17%), and significantly more zippers (Figure S5G; 11%–79%) in the double knockouts compared to controls. Finally, a direct comparison of the double knockouts with the *Flt4* single knockouts (Figures S5H and S5I) revealed that there were no biologically significant differences between these two models. Altogether, these data show that the inability of *Flt4*^*iLECKO*^ mice to form button junctions is not due to increased activation of VEGFR2.

### Overexpression of the NOTCH1 intracellular domain rescues button formation

**Figure 4.**
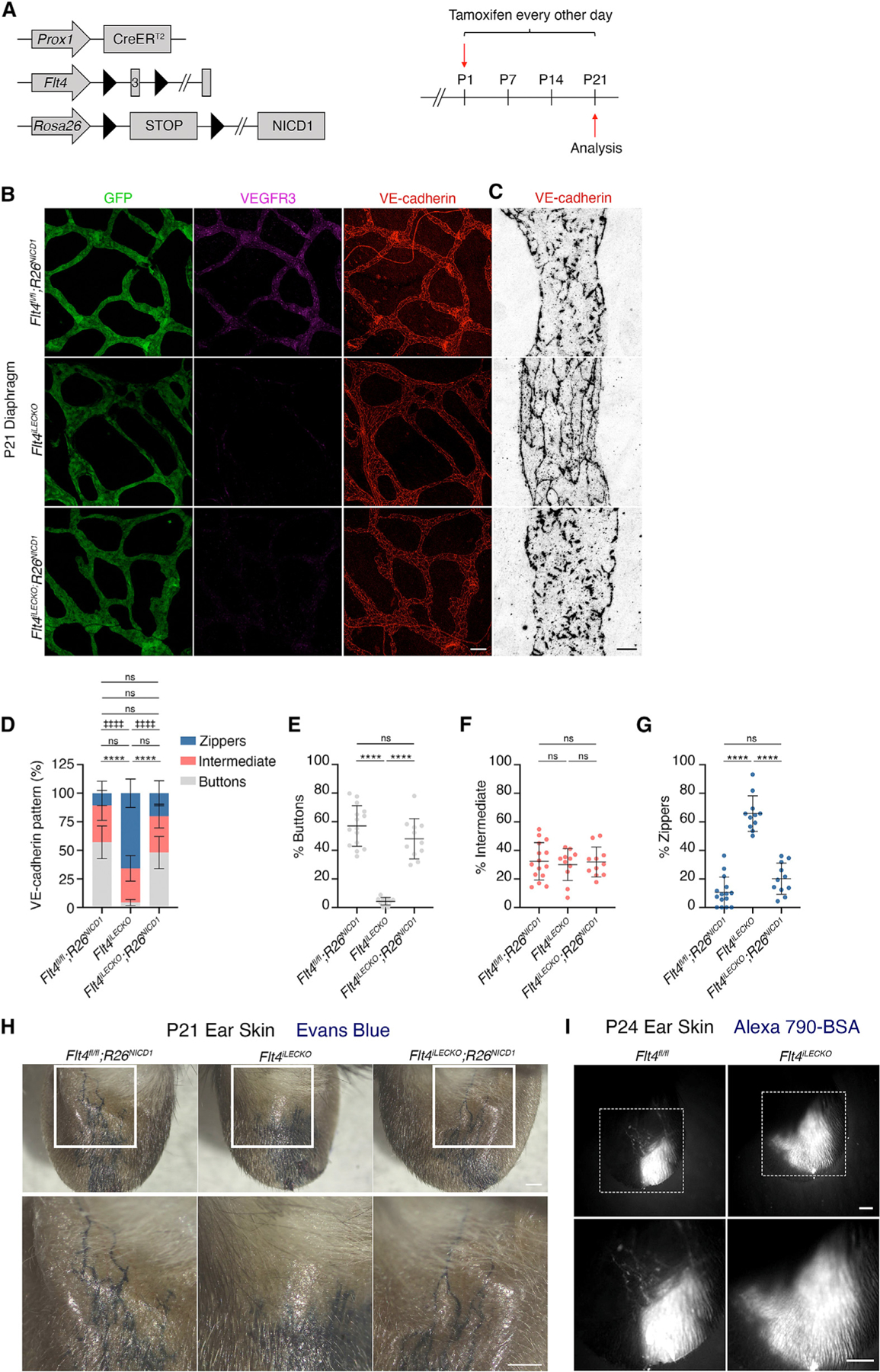
Overexpression of the NOTCH1 intracellular domain (NICD1) rescues button formation and lymphatic function in the absence of VEGFR3 (A) Tamoxifen procedure for deletion of *Flt4* and induction of constitutive *NICD1* expression. (B) Lymphatic vessels in P21 diaphragms were immunostained for GFP (green), VEGFR3 (magenta), and VE-cadherin (red). (C) Higher magnification images of VE-cadherin immunostaining at lymphatic endothelial intercellular junctions. (D) Quantification of the three different junction types in *Flt4*^*fl/fl*^*;R26*^*NICD1*^ (N = 5 mice; n = 15 FOVs), *Flt4*^*iLECKO*^ (N = 4 mice; n = 11 FOVs), and *Flt4*^*iLECKO*^*;R26*^*NICD1*^ (N = 4 mice; n = 11 FOVs) mice. (E–G) Individual graphs of button, intermediate and zipper junctions presented in (D). (H) Evans blue dye was injected into the ears of *Flt4*^*fl/fl*^*;R26*^*NICD1*^, *Flt4*^*iLECKO*^, and *Flt4*^*iLECKO*^*;R26*^*NICD1*^ mice at P21, and dye uptake into the lymphatic vasculature was imaged. (I) BSA conjugated to Alexa 790 was injected into P24 mouse ears and imaged. Two-way ANOVA with Sidak’s post hoc test. ****p < 0.0001 buttons; ‡‡‡‡p < 0.0001 zippers; ns, non-significant. All data are presented as mean ± SD. Scale bar in (B) represents 50 μm, in (C) represents 10 μm, in (H) represents 500 μm, and in (I) represents 1 mm. FOVs, fields of view.

Having shown that loss of VEGFR3 reduces the expression of NOTCH1 and thereby decreases NICD1 cleavage in hdLECs, we hypothesized that overexpression of the active NICD1 fragment could restore NOTCH signaling and rescue button formation in *Flt4*^*iLECKO*^ mice. Thus, we bred *Flt4*^*iLECKO*^ mice with Rosa26-NICD1 mice (hereafter: *Flt4*^*iLECKO*^*;R26*^*NICD1*^) that express the NOTCH1 NICD, which induces constitutive NOTCH1 signaling after Cre recombination^21^ (Figure 4A). As before, when we stained for VE-cadherin, we observed predominantly button junctions in *Flt4*^*fl/fl*^*;R26*^*NICD1*^ control diaphragms that lacked the Cre allele but almost no button junctions in the *Flt4*^*iLECKO*^ diaphragms (Figures 4B and 4C; 60% vs. 4%). However, in the *Flt4*^*iLECKO*^*;R26*^*NICD1*^ mice, button junctions accounted for 48% of the junction type, reminiscent of the control mice (Figures 4B and 4C). Quantification of the junction types indicated a complete rescue of button junction formation in the *Flt4*^*iLECKO*^*;R26*^*NICD1*^ mice, whose junction types did not significantly differ from the control mice (Figures 4D–4G). Together, these data indicate that NOTCH1 receptor signaling is sufficient to completely rescue button formation in the absence of VEGFR3 signaling.

### Lymphatic capillary function is impaired by loss of VEGFR3 and can be rescued by NICD overexpression

Since the *Flt4*^*iLECKO*^ mice fail to form button junctions in the diaphragm and ear skin (Figures 1 and 2), we investigated whether this would correlate with a decrease in fluid uptake by the lymphatic capillaries. Evans blue dye was injected into the tip of the mouse ear where the lymphatic capillaries are located and immediately filled lymphatic vessels with the dye in *Flt4*^*fl/fl*^*; R26*^*NICD1*^ control mice (Figures 4H and 4I). Even though postnatal deletion of VEGFR3 caused lymphatic hyperplasia in the ear lymphatic vasculature in *Flt4*^*iLECKO*^ mice (Figure S2), as previously reported,^13^ Evans blue dye injected into the ear skin of *Flt4*^*iLECKO*^ mice still did not fill any vascular structures (Figures 4H and 4I), indicating that the zipper junctions in these mice prevents dye absorption. Because overexpression of NICD1 rescued button junction formation in *Flt4*^*iLECKO*^ mice, we tested whether this would translate into a functional rescue of interstitial absorption. When injected into the edge of the ear skin of *Flt4*^*iLECKO*^;*R26*^*NICD1*^ mice, the lymphatic vessels became filled with Evans blue dye, indicating that the overexpression of NICD1 is sufficient to rescue both button junction formation and function (Figure 4H). To confirm the Evans blue dye findings, we conjugated the macromolecule BSA to the near infrared fluorophore Alexa 790 and injected it into the mouse ear. As before, the BSA filled the lymphatic vessels in the *Flt4*^*fl/fl*^ controls, while it filled only the interstitium in the *Flt4*^*iLECKO*^ ears (Figure 4I).

### NICD1 overexpression accelerates button junction formation

Our findings regarding the ability of Notch signaling to rescue button formation and function led us to investigate whether constitutive expression of NICD1 alone could accelerate or enhance button junction formation in otherwise normal mice. Tamoxifen was injected at P1 to induce recombination in *Prox1CreER*^*T2*^*;R26*^*NICD1*^ and *R26*^*NICD1*^ controls. In P14 diaphragms, we found that *Prox1CreER*^*T2*^*;R26*^*NICD1*^ mice had significantly more button junctions compared to control mice (59% vs. 32%, Figures S6C–S6F). However, P21 diaphragms showed no significant difference in button or zipper junctions between the *Prox1CreER*^*T2*^*;R26*^*NICD1*^ and R26NICD1 controls (Figures S6G, S6H, and S6I–S6L). Thus, these data suggest that NOTCH1 is sufficient to accelerate the formation of button junctions, but it does not induce the formation of more button junctions than normally observed at P21.

## DISCUSSION

This study demonstrates that VEGFR3 is required for button junction formation and interstitial fluid absorption in multiple tissues. Because genetic deletion of VEGFR3 at a developmental time point when buttons are fully formed does not affect the number or morphology of buttons, we conclude that VEGFR3 signaling does not maintain button junctions and, further, that there is a critical postnatal developmental window wherein VEGFR3 must be activated to successfully form buttons. To investigate the mechanisms downstream of VEGFR3 activation, we cultured hdLECs with a *FLT4* knockdown lentivirus and found that NOTCH1 expression and cleavage was inhibited. We were able to fully rescue the formation of button junctions to control levels by overexpressing the constitutively active NOTCH1 intracellular domain (NICD1) in the *Flt4*^*iLECKO*^ mice. The NICD1 fragment also rescued the ability of lymphatic capillaries to absorb interstitial tracer injected into the ear. Finally, our data show that NOTCH1 signaling can accelerate button formation, so NOTCH1 should be investigated in future studies as a potential inducer of button formation.

### VEGFR signaling interacts with the NOTCH pathway

While previous studies have demonstrated that VEGFR3 and NOTCH receptor signaling are closely linked, a direct role for these specific receptors in button formation has not previously been demonstrated. VEGF-A/VEGFR2 signaling was shown to induce DLL4-NOTCH signaling during angiogenic sprouting,^22^ although these findings were challenged by studies suggesting that VEGFR2 only weakly activates DLL4 and instead proposed a role for VEGFR3 activating DLL4 and NOTCH signaling.^23,24^ In lymphatics, loss of VEGFR3 was shown to decrease DLL4 expression, which in turn decreased NOTCH1 signaling.^13^ Surprisingly, we found that DLL4 levels did not change at the protein level, although NOTCH1 activation was significantly decreased in *FLT4* knockdown hdLECs. Importantly, we show that forced activation of NOTCH1 signaling in the absence of VEGFR3 restores the ability of lymphatic capillaries to form buttons and absorb interstitial fluid, identifying NOTCH1 as a potential druggable target even in the absence of VEGFR3. Future studies will be needed to probe which NOTCH ligands are inducing NOTCH1 cleavage and whether NOTCH ligands are tissue specific in the context of button formation.

### Relevance of the findings to congenital and acquired lymphedema

Heterozygous point mutations in the *FLT4* gene cause the most common type of congenital lymphedema, called Milroy disease.^25,26^ Milroy patients completely fail to absorb interstitial tracer during a lymphoscintigraphy exam, similar to our *Flt4*^*iLECKO*^ mice here.^27^ Given the known role of VEGFR3 as the main receptor required for lymphangiogenesis,^28^ it was first suspected that dermal lymphatic vessel aplasia caused Milroy disease.^29^ However, this idea was challenged when skin biopsies from the feet of Milroy patients were found to contain lymphatic vessels.^27^ Most mutations in *FLT4* occur in the kinase domain, thereby abolishing the kinase activity of VEGFR3.^30^ Because VEGFR3 makes homodimers or heterodimers with VEGFR2 to signal, these point mutations act in a dominant-negative manner.^31,32^ The lack of a phenotype in the *Kdr* knockouts here argues against heterodimers being involved in button formation. Mice with a similar point mutation in *Flt4*, known as Chy mice, are a mouse model of congenital lymphedema.^29^ Thus, the *Flt4*^*iLECKO*^ mice reported here that lack VEGFR3 protein likely mimic the dominant-negative mutations in the Milroy patients or Chy mice that have very low residual VEGFR3 signaling. Based on our present data, we hypothesize that a failure to form buttons may underlie the pathogenesis of the Milroy patients and that future work is needed to investigate our signaling mechanism in the Chy mice.

Angiopoietin 2 is also required for button formation,^5^ and mutations in ANGPT2 have since been identified in congenital lymphedema patients.^33^ Interestingly, a recent study found that inhibition of angiopoietin 2 or deletion of the angiopoietin receptors, *Tie1* and *Tie2*, decreased the expression of VEGFR3 in LECs.^34^ However, to our knowledge, lymphoscintigraphy was not performed on these patients, so it is unknown whether they fail to absorb interstitial tracer and thus if defective button formation contributes to this form of lymphedema. Together with the findings here, it argues that a single signaling pathway, namely ANGPT2/VEGFR3/NOTCH1, is responsible for regulating button junction formation.

While congenital lymphedema is caused by genetic mutations, acquired lymphedema develops after injury to the lymphatic vasculature.^35^ A recent study found that acquired lymphedema patients have elevated levels of the proinflammatory mediator leukotriene B_4_ (LTB_4_),^36^ which inhibited VEGFR3 expression and phosphorylation and inhibited NOTCH signaling in LECs. Thus, it is tantalizing to speculate that the lymphatic capillaries of acquired lymphedema patients may have aberrantly zippered junctions as part of the disease progression. Further supporting our data, Tian et al. also showed that lymphatic-specific deletion of *Notch1* prevented the absorption of an interstitial tracer and prevented a pharmacological rescue of tail lymphedema, indicating that NOTCH1 signaling must remain intact for LTB_4_ inhibition to be effective at preventing lymphedema. Given that we show here that overactivation of NOTCH1 signaling both rescues the button defect in *Flt4*^*iLECKO*^ mice and accelerates button formation in healthy mice, it is likely that the deletion of *Notch1* phenocopies the *Flt4*^*iLECKO*^ mice and prevents button formation, but this remains to be tested in future studies.

### Limitations of the study

While we show here that VEGFR3 is needed for postnatal button formation and is dispensable for maintaining buttons, our study has a few limitations. One limitation is that we show that NOTCH1 NICD is capable of rescuing button formation in the absence of VEGFR3, but we have not determined whether NOTCH1 is required for normal button junction formation. We will need to conduct future studies using *Notch1*^*fl/fl*^ conditional knockout mice to answer this question. Another limitation extends to extrapolating our findings here to the Milroy patients. Here, we are deleting both alleles of the *Flt4* gene, leading to a complete loss of protein and signaling, whereas the Milroy patients commonly harbor a point mutation in the kinase domain of *Flt4* that acts in a dominant-negative manner.^30^ Although the patients have a severe loss of VEGFR3 signaling, there is likely some residual signaling remaining. Future studies using the Chy mouse strain will be needed to confirm our findings here regarding defective button junction formation.

### Conclusions

In summary, our data show that VEGFR3 is required for button junction formation in lymphatic capillaries and interstitial fluid absorption. We were able to rescue this defect by overexpressing NICD1 to enhance NOTCH1 signaling, restoring lymphatic function in these mice. Finally, we show that overexpression of NICD1 can accelerate button junction formation, suggesting that NOTCH1 may be an important inducer of button junction formation developmentally.

## STAR★METHODS

### RESOURCE AVAILABILITY

#### Lead contact

Further information and requests for resources and reagents should be directed to and will be fulfilled by the lead contact, Josh Scallan (jscallan@usf.edu).

#### Materials availability

Upon completion of a Material Transfer Agreement, we will share any reagents.

#### Data and code availability

All data reported in this paper will be shared by the lead contact upon request. This paper does not report original code. Any additional information required to reanalyze the data reported in this paper is available from the lead contact upon request.

### EXPERIMENTAL MODEL AND STUDY PARTICIPANT DETAILS

#### Mice

Mouse strains used in this study include *Prox1CreER*^*T2*^, *Prox1GFP,* and *Flt4*^*fl/fl*^.^9,10,14^ The *R26*^*NICD1*^ strain (Strain#: 008159) and the *Kdr*^*fl/fl*^ strain (Strain#: 018977) were purchased from The Jackson Laboratory.^21,37^ Both male and female mice were used on a C57BL/6J background and displayed no sex differences in phenotypes, but individual mice were not sexed. Mice had *ad libitum* access to food and water. To breed the final mice used in this study, male mice expressing *Prox1CreER*^*T2*^ along with a homozygous floxed gene were crossed with females expressing only the homozygous floxed gene without *Prox1CreER*^*T2*^. One parent expressed a heterozygous *Prox1GFP* allele or *R26-NICD* allele. In this manner, littermate controls and knockouts were stained and quantified together but not every pup harbored the Prox1GFP allele. To delete genes at birth, pups were injected subcutaneously with TM (100 μg in5 μL of sunflower oil) from postnatal day P1 and then every other day after until analysis at P21. To delete genes in weanlings, mice were injected intraperitoneally (1 mg in 50 μL of sunflower oil) starting at P21 and then every other day after until analysis at P35. In experiments with the *Prox1CreER*^*T2*^*;R26-NICD1* mice, tamoxifen was injected at P1 and P3, as previously published.^11^ All experiments were performed in accordance with the University of South Florida guidelines and were approved by the institutional IACUC committee.

#### Cell lines and shRNA

Primary human dermal lymphatic endothelial cells (hdLECs, PromoCell, male donors) were cultured on fibronectin-coated plates using EBM-2 media (PromoCell). All hdLECs were used at passage 6–7 for shRNA knockdown. Cells were infected with either a scrambled control (Scr) or an shRNA targeting *FLT4* (shFLT4) (sequence: GAGAGACTTTGAGCAGCCATT)^38^ for 48 h (VectorBuilder). The virus was removed, and the cells grown for a further 48 h before collection of either protein or RNA.

### METHOD DETAILS

#### Evans blue assay

Mice were anesthetized with ketamine and placed prone on an acrylic board. A 2% solution of Evans blue dye and 2% BSA diluted in sterile saline was freshly prepared prior to the experiment. Approximately 2 μL of the Evans Blue dye was injected into the center edge of the ear using a glass needle attached to a syringe. The glass needle was fabricated by pulling capillary glass on a vertical puller (Narishige, #PC-10) before grinding the tip to a diameter of approximately 100 μm. Images of the dye-filled lymphatics in the ear were captured using a color camera (Zeiss Axiocam 208 Color) mounted on a dissection microscope (Zeiss Stemi 508).

#### Near infrared fluorescence imaging

Bovine serum albumin (BSA) was conjugated to Alexa 790 and purified through centrifugal molecular weight cutoff filters as previously described.^39^ Mice were anesthetized with ketamine and placed prone on an acrylic board. A 25 μL glass syringe with a 30-gauge needle was used to inject 2 μL of the dye conjugate (~500 μg BSA total) into the mouse ear of P24 mice. Ears were immediately imaged on an Olympus MVX10 fluorescence stereomicroscope outfitted with a Photometrics Evolve 512 Delta EMCCD camera controlled by μManager open source software.

#### Whole-mount immunostaining

Whole-mount immunostaining was performed as described previously.^11^ Unless otherwise stated, all procedures were performed at 4°C on an orbital shaker (Belly Dancer, IBI Scientific). Briefly, tissues were fixed overnight with 2% PFA in PBS and then washed with PBS before permeabilizing for 1 h with PBS +0.3% Triton X-100 (PBST). Tissues were blocked for 2 h with 3% donkey serum in PBST and incubated overnight with primary antibodies diluted in PBST. After five 15-min washes with PBST, tissues were incubated with secondary antibodies diluted in PBST for 2 h on an orbital shaker at room temperature. Following five 15-min washes in PBST, the tissues were incubated with DAPI (Sigma) at room temperature for 5 min and then washed in PBS. Tissues were then mounted onto glass slides (Superfrost plus microscope slides, Fisherbrand) using ProLong Diamond Antifade Mountant (Invitrogen) and stored at 4°C. Details of primary and secondary antibodies are outlined in the resource table. Images were acquired using a 40x water immersion objective on a Leica SP8 confocal microscope and analyzed with FIJI software (NIH). Figures were created using Adobe Photoshop.

#### Quantification of VE-cadherin junction types

The junction types were quantified using FIJI software from images of lymphatic capillaries. Three types of junctions have been defined – buttons, intermediate, and zipper junctions. We quantified each junction type by measuring junction lengths and then normalizing as a percentage of total length as previously published.^5,7^ Buttons were considered short, discrete, unconnected sections of VE-cadherin protein that were perpendicular to the cell membrane. To objectively distinguish between button, intermediate and zipper junctions, we measured and averaged the length of each junction type from 3 samples, allowing us to define a button junction as <4.72 μm in length, intermediate junction as >4.72 μm but <7.85 μm in length, and a zipper junction as >7.85 μm in length. Using these criteria, the total length for each junction type was calculated as a percentage of total junction length.

#### Western blot

Total protein was collected from hdLECs lysed with RIPA buffer (Pierce, ThermoFisher Scientific) and the protein concentration was measured with a BCA protein assay kit (Pierce, ThermoFisher Scientific). Gel electrophoresis was performed using the mini gel tank (Invitrogen), the iBlot 2 dry blotting system (Invitrogen), and the iBind automated western system (Invitrogen). Protein was visualized using SuperSignal west pico plus chemiluminescent substrate (ThermoFisher Scientific).

#### RNA isolation and qRT-PCR

Total RNA was collected from hdLECs according to manufacturer’s instructions in the RNeasy plus mini kit (Qiagen). cDNA was synthesized according to manufacturer’s instructions in the Advantage RT-for-PCR kit (Tala). The QuantStudio 6 Pro Realtime System (Applied Biosystems) was used to carry out quantitative RT-PCR using Taqman probes (ThermoFisher). The cycle threshold (Ct) value for GAPDH was used to normalize the Ct value for each gene of interest.

### QUANTIFICATION AND STATISTICAL ANALYSIS

All junction quantification was analyzed by two-way ANOVA with Sidak’s post hoc test. All qRT-PCR data were analyzed by unpaired Student’s t-test. All graphical data are presented mean ± standard deviation, except for Figure 3, which is presented as mean ± SEM.

## Supplementary Material

1

## Figures and Tables

**Table:** KEY RESOURCES TABLE

REAGENT or RESOURCE	SOURCE	IDENTIFIER
Antibodies		

rat anti-VE-cadherin	BD Pharmingen	550548; RRID: AB_2244723
rabbit anti-PROX1	Abcam	ab101851; RRID:AB_10712211
goat anti-PROX1	R&D Systems	AF2727; RRID:AB_2170716
Alexa Fluor 488-conjugated anti-GFP	Life Technologies	A21311; RRID:AB_221477
goat anti-VEGFR3	R&D Systems	AF743; RRID:AB_355563
goat anti-VEGFR2	R&D Systems	AF644; RRID:AB_355500
rabbit anti-VEGFR2	ThermoFisher Scientific	MA5–15157; RRID:AB_10986085
rabbit anti-LYVE1	AngioBio	11–034; RRID:AB_2813732
Alexa Fluor 488 donkey anti-rabbit IgG (H + L)	Invitrogen	A–21206; RRID:AB_2535792
Alexa Fluor 594 donkey anti-rat IgG (H + L)	Invitrogen	A–21209; RRID:AB_2535795
Alexa Fluor 647 donkey anti-goat IgG (H + L)	Invitrogen	A–21447; RRID:AB_141844
goat anti-VEGFR3	R&D Systems	AF349; RRID:AB_355314
rabbit anti-VEGFR2	Cell Signaling	2479; RRID:AB_2212507
rabbit anti-phospho-VEGFR2 Tyr1175	Cell Signaling	2478; RRID:AB_331377
rabbit anti-cleaved NOTCH1	Cell Signaling	4147; RRID:AB_2153348
Rabbit anti-cleaved NOTCH4	Millipore Sigma	SAB4502023; RRID:AB_10744463
mouse anti-β-actin	Cell Signaling	3700; RRID:AB_2242334
donkey anti-rabbit IgG (H + L) Highly Cross-Adsorbed Secondary Antibody, HRP	Invitrogen	A16035; RRID:AB_2534709
donkey anti-goat IgG (H + L) Cross-Adsorbed Secondary Antibody, HRP	Invitrogen	A16005; RRID:AB_2534679
donkey anti-mouse IgG (H + L) Highly Cross-Adsorbed Secondary Antibody, HRP	Invitrogen	A16017; RRID:AB_2534691

Chemicals, peptides, and recombinant proteins		

Human fibronectin	Millipore Sigma	FC010
Tamoxifen	Millipore Sigma	T5648
Evans blue	Millipore Sigma	E2129
Sunflower oil	Millipore Sigma	S5007
RIPA buffer	Millipore Sigma	20–188

Critical commercial assays		

Pierce BCA Protein Assay Kit	ThermoFisher Scientific	23227
RNeasy Plus Mini Kit	Qiagen	74134
iBind Cards	Invitrogen	SLF1010
iBind Flex. Solution Kit	Invitrogen	SLF2020
SuperSignal West Pico PLUS Chemiluminescent Substrate	ThermoScientific	34580

Experimental models: Cell lines		

Primary human dermal lymphatic endothelial cells	PromoCell	12216

Experimental models: Organisms/strains		

*Prox1CreER* ^*T2*^	Bazigou et al.^10^	RRID:MGI:6438646
*Prox1GFP*	Choi et al.^14^	RRID:MGI:5004059
*Flt4* ^*fl/fl*^	Keller et al.^9^	N/A
*R26* ^*NICD1*^	Murtaugh et al.^21^ The Jackson Laboratory	Strain#: 008159; RRID:MGI:2684336
*Kdr* ^*fl/fl*^	Hooper et al.^37^ The Jackson Laboratory	Strain#: 018977; RRID:IMSR_JAX:018977

Recombinant DNA		

pLV[shRNA]-EGFP:T2A:Puro-U6>Scramble_shRNA	This paper	N/A
pLV[shRNA]-EGFP-U6>TTC25[shRNA]	This paper	N/A

Software and algorithms		

FIJI	NIH	https://imagej.net/software/fiji/; RRID:SCR_002285
Prism V9	GraphPad Software	https://www.graphpad.com/scientific-software/prism/; RRID:SCR_002798
Adobe Photoshop	Adobe	https://www.adobe.com/products/photoshop.html; RRID:SCR_014199

## Highlights

- VEGFR3 is required for the development of lymphatic capillary button junctions
- VEGFR3 is not required for maintaining button junctions after their formation
- Failure to form buttons impedes the absorption of interstitial molecules
- NOTCH1 signaling can rescue button formation and function in the absence of VEGFR3

## References

[1] Brouillard P, Witte MH, Erickson RP, Damstra RJ, Becker C, Quéré I, and Vikkula M (2021). Primary lymphoedema. Nat. Rev. Dis. Primers 7, 77. 10.1038/s41572-021-00309-7. 34675250

[2] Iyer D, Jannaway M, Yang Y, and P Scallan J (2020). Lymphatic valves and lymph flow in cancer-related lymphedema. Cancers 12, 2297. 10.3390/cancers12082297. 32824219 PMC7464955

[3] Baluk P, Fuxe J, Hashizume H, Romano T, Lashnits E, Butz S, Vestweber D, Corada M, Molendini C, Dejana E, and McDonald DM (2007). Functionally specialized junctions between endothelial cells of lymphatic vessels. J. Exp. Med 204, 2349–2362. 10.1084/jem.20062596. 17846148 PMC2118470

[4] Yao LC, Baluk P, Srinivasan RS, Oliver G, and McDonald DM (2012). Plasticity of button-like junctions in the endothelium of airway lymphatics in development and inflammation. Am. J. Pathol 180, 2561–2575. 10.1016/j.ajpath.2012.02.019. 22538088 PMC3378913

[5] Zheng W, Nurmi H, Appak S, Sabine A, Bovay E, Korhonen EA, Orsenigo F, Lohela M, D’Amico G, Holopainen T, (2014). Angiopoietin 2 regulates the transformation and integrity of lymphatic endothelial cell junctions. Genes Dev 28, 1592–1603. 10.1101/gad.237677.114. 25030698 PMC4102766

[6] Bernier-Latmani J, Cisarovsky C, Demir CS, Bruand M, Jaquet M, Davanture S, Ragusa S, Siegert S, Dormond O, Benedito R, (2015). DLL4 promotes continuous adult intestinal lacteal regeneration and dietary fat transport. J. Clin. Invest 125, 4572–4586. 10.1172/JCI82045. 26529256 PMC4665794

[7] Zhang F, Zarkada G, Han J, Li J, Dubrac A, Ola R, Genet G, Boyé K, Michon P, Künzel SE, (2018). Lacteal junction zippering protects against diet-induced obesity. Science 361, 599–603. 10.1126/science.aap9331. 30093598 PMC6317738

[8] Churchill MJ, du Bois H, Heim TA, Mudianto T, Steele MM, Nolz JC, and Lund AW (2022). Infection-induced lymphatic zippering restricts fluid transport and viral dissemination from skin. J. Exp. Med 219, e20211830. 10.1084/jem.20211830. 35353138 PMC8972184

[9] Keller TCS 4th, Lim L, Shewale SV, McDaid K, Martí-Pàmies Í, Tang AT, Wittig C, Guerrero AA, Sterling S, Leu NA, (2021). Genetic blockade of lymphangiogenesis does not impair cardiac function after myocardial infarction. J. Clin. Invest 131, e147070. 10.1172/JCI147070. 34403369 PMC8516448

[10] Bazigou E, Lyons OTA, Smith A, Venn GE, Cope C, Brown NA, and Makinen T (2011). Genes regulating lymphangiogenesis control venous valve formation and maintenance in mice. J. Clin. Invest 121, 2984–2992. 10.1172/JCI58050. 21765212 PMC3223924

[11] Yang Y, Cha B, Motawe ZY, Srinivasan RS, and Scallan JP (2019). VE-cadherin is required for lymphatic valve formation and maintenance. Cell Rep 28, 2397–2412.e4. 10.1016/j.celrep.2019.07.072. 31461654 PMC6743082

[12] Scallan JP, Knauer LA, Hou H, Castorena-Gonzalez JA, Davis MJ, and Yang Y (2021). Foxo1 deletion promotes the growth of new lymphatic valves. J. Clin. Invest 131, e142341. 10.1172/JCI142341. 34263740 PMC8279588

[13] Zhang Y, Ulvmar MH, Stanczuk L, Martinez-Corral I, Frye M, Alitalo K, and Mӓkinen T (2018). Heterogeneity in VEGFR3 levels drives lymphatic vessel hyperplasia through cell-autonomous and non-cell-autonomous mechanisms. Nat. Commun 9, 1296. 10.1038/s41467-018-03692-0. 29615616 PMC5882855

[14] Choi I, Chung HK, Ramu S, Lee HN, Kim KE, Lee S, Yoo J, Choi D, Lee YS, Aguilar B, and Hong YK (2011). Visualization of lymphatic vessels by Prox1-promoter directed GFP reporter in a bacterial artificial chromosome-based transgenic mouse. Blood 117, 362–365. 10.1182/blood-2010-07-298562. 20962325 PMC3037757

[15] Sabine A, Bovay E, Demir CS, Kimura W, Jaquet M, Agalarov Y, Zangger N, Scallan JP, Graber W, Gulpinar E, (2015). FOXC2 and fluid shear stress stabilize postnatal lymphatic vasculature. J. Clin. Invest 125, 3861–3877. 10.1172/JCI80454. 26389677 PMC4607114

[16] Shawber CJ, Funahashi Y, Francisco E, Vorontchikhina M, Kitamura Y, Stowell SA, Borisenko V, Feirt N, Podgrabinska S, Shiraishi K, (2007). Notch alters VEGF responsiveness in human and murine endothelial cells by direct regulation of VEGFR-3 expression. J. Clin. Invest 117, 3369–3382. 10.1172/JCI24311. 17948123 PMC2030453

[17] Zheng W, Tammela T, Yamamoto M, Anisimov A, Holopainen T, Kaijalainen S, Karpanen T, Lehti K, Ylӓ-Herttuala S, and Alitalo K (2011). Notch restricts lymphatic vessel sprouting induced by vascular endothelial growth factor. Blood 118, 1154–1162. 10.1182/blood-2010-11-317800. 21566091

[18] Gale NW, Dominguez MG, Noguera I, Pan L, Hughes V, Valenzuela DM, Murphy AJ, Adams NC, Lin HC, Holash J, (2004). Haploinsufficiency of delta-like 4 ligand results in embryonic lethality due to major defects in arterial and vascular development. Proc. Natl. Acad. Sci. USA 101, 15949–15954. 10.1073/pnas.0407290101. 15520367 PMC524697

[19] Muley A, Kim Uh M, Salazar-De Simone G, Swaminathan B, James JM, Murtomaki A, Youn SW, McCarron JD, Kitajewski C, Gnarra Buethe M, (2022). Unique functions for Notch4 in murine embryonic lymphangiogenesis. Angiogenesis 25, 205–224. 10.1007/s10456-021-09822-5. 34665379 PMC9054879

[20] D’Souza B, Meloty-Kapella L, and Weinmaster G (2010). Canonical and non-canonical Notch ligands. Curr. Top. Dev. Biol 92, 73–129. 10.1016/S0070-2153(10)92003-6. 20816393 PMC4286395

[21] Murtaugh LC, Stanger BZ, Kwan KM, and Melton DA (2003). Notch signaling controls multiple steps of pancreatic differentiation. Proc. Natl. Acad. Sci. USA 100, 14920–14925. 10.1073/pnas.2436557100. 14657333 PMC299853

[22] Hellstrӧm M, Phng LK, Hofmann JJ, Wallgard E, Coultas L, Lindblom P, Alva J, Nilsson AK, Karlsson L, Gaiano N, (2007). Dll4 signalling through Notch1 regulates formation of tip cells during angiogenesis. Nature 445, 776–780. 10.1038/nature05571. 17259973

[23] Tammela T, Zarkada G, Nurmi H, Jakobsson L, Heinolainen K, Tvorogov D, Zheng W, Franco CA, Murtomӓki A, Aranda E, (2011). VEGFR-3 controls tip to stalk conversion at vessel fusion sites by reinforcing Notch signalling. Nat. Cell Biol 13, 1202–1213. 10.1038/ncb2331. 21909098 PMC3261765

[24] Benedito R, Rocha SF, Woeste M, Zamykal M, Radtke F, Casanovas O, Duarte A, Pytowski B, and Adams RH (2012). Notch-dependent VEGFR3 upregulation allows angiogenesis without VEGF-VEGFR2 signalling. Nature 484, 110–114. 10.1038/nature10908. 22426001

[25] Ferrell RE, Levinson KL, Esman JH, Kimak MA, Lawrence EC, Barmada MM, and Finegold DN (1998). Hereditary lymphedema: evidence for linkage and genetic heterogeneity. Hum. Mol. Genet 7, 2073–2078. 10.1093/hmg/7.13.2073. 9817924

[26] Irrthum A, Karkkainen MJ, Devriendt K, Alitalo K, and Vikkula M (2000). Congenital hereditary lymphedema caused by a mutation that inactivates VEGFR3 tyrosine kinase. Am. J. Hum. Genet 67, 295–301. 10.1086/303019. 10856194 PMC1287178

[27] Mellor RH, Hubert CE, Stanton AWB, Tate N, Akhras V, Smith A, Burnand KG, Jeffery S, Mӓkinen T, Levick JR, and Mortimer PS (2010). Lymphatic dysfunction, not aplasia, underlies Milroy disease. Microcirculation 17, 281–296. 10.1111/j.1549-8719.2010.00030.x. 20536741

[28] Jeltsch M, Kaipainen A, Joukov V, Meng X, Lakso M, Rauvala H, Swartz M, Fukumura D, Jain RK, and Alitalo K (1997). Hyperplasia of lymphatic vessels in VEGF-C transgenic mice. Science 276, 1423–1425. 10.1126/science.276.5317.1423. 9162011

[29] Karkkainen MJ, Saaristo A, Jussila L, Karila KA, Lawrence EC, Pajusola K, Bueler H, Eichmann A, Kauppinen R, Kettunen MI, (2001). A model for gene therapy of human hereditary lymphedema. Proc. Natl. Acad. Sci. USA 98, 12677–12682. 10.1073/pnas.221449198. 11592985 PMC60113

[30] Liu N, and Gao M (2021). FLT4 mutations are associated with segmental lymphatic dysfunction and initial lymphatic aplasia in patients with Milroy disease. Genes 12, 1611. 10.3390/genes12101611. 34681005 PMC8535675

[31] Gordon K, Spiden SL, Connell FC, Brice G, Cottrell S, Short J, Taylor R, Jeffery S, Mortimer PS, Mansour S, and Ostergaard P (2013). FLT4/VEGFR3 and Milroy disease: novel mutations, a review of published variants and database update. Hum. Mutat 34, 23–31. 10.1002/humu.22223. 23074044

[32] Karkkainen MJ, Ferrell RE, Lawrence EC, Kimak MA, Levinson KL, McTigue MA, Alitalo K, and Finegold DN (2000). Missense mutations interfere with VEGFR-3 signalling in primary lymphoedema. Nat. Genet 25, 153–159. 10.1038/75997. 10835628

[33] Leppӓnen VM, Brouillard P, Korhonen EA, Sipilӓ T, Jha SK, Revencu N, Labarque V, Fastré E, Schlӧgel M, Ravoet M, (2020). Characterization of ANGPT2 mutations associated with primary lymphedema. Sci. Transl. Med 12, eaax8013. 10.1126/scitranslmed.aax8013. 32908006

[34] Korhonen EA, Murtomaki A, Jha SK, Anisimov A, Pink A, Zhang Y, Stritt S, Liaqat I, Stanczuk L, Alderfer L, (2022). Lymphangiogenesis requires Ang2/Tie/PI3K signaling for VEGFR3 cell surface expression. J. Clin. Invest 10.1172/JCI155478. PMC9337826 35763346

[35] Rockson SG (2021). Advances in Lymphedema. Circ. Res 128, 2003–2016. 10.1161/CIRCRESAHA.121.318307. 34110905

[36] Tian W, Rockson SG, Jiang X, Kim J, Begaye A, Shuffle EM, Tu AB, Cribb M, Nepiyushchikh Z, Feroze AH, (2017). Leukotriene B4 antagonism ameliorates experimental lymphedema. Sci. Transl. Med 9, eaal3920. 10.1126/scitranslmed.aal3920. 28490670

[37] Hooper AT, Butler JM, Nolan DJ, Kranz A, Iida K, Kobayashi M, Kopp HG, Shido K, Petit I, Yanger K, (2009). Engraftment and reconstitution of hematopoiesis is dependent on VEGFR2-mediated regeneration of sinusoidal endothelial cells. Cell Stem Cell 4, 263–274. 10.1016/j.stem.2009.01.006. 19265665 PMC3228275

[38] Heinolainen K, Karaman S, D’Amico G, Tammela T, Sormunen R, Eklund L, Alitalo K, and Zarkada G (2017). VEGFR3 modulates vascular permeability by controlling VEGF/VEGFR2 signaling. Circ. Res 120, 1414–1425. 10.1161/CIRCRESAHA.116.310477. 28298294 PMC6959003

[39] Jannaway M, and Scallan JP (2021). VE-cadherin and vesicles differentially regulate lymphatic vascular permeability to solutes of various sizes. Front. Physiol 12, 687563. 10.3389/fphys.2021.687563. 34621180 PMC8491776

