# Supplementary material for: VEGFR3 is required for button junction formation in lymphatic vessels
Source: Cell Rep. Author manuscript; Available in PMC 2023 Sep 15. (PMC10503778; doi:10.1016/j.celrep.2023.112777)
Supplement: 1 [file NIHMS1920505-supplement-1.pdf]

**Cell Reports, Volume *42***

## Supplemental information

# VEGFR3 is required for button junction formation in lymphatic vessels

**Melanie Jannaway, Drishya Iyer, Diandra M. Mastrogiacomo, Kunyu Li, Derek C. Sung, Ying Yang, Mark L. Kahn, and Joshua P. Scallan**

## SUPPLEMENTAL INFORMATION

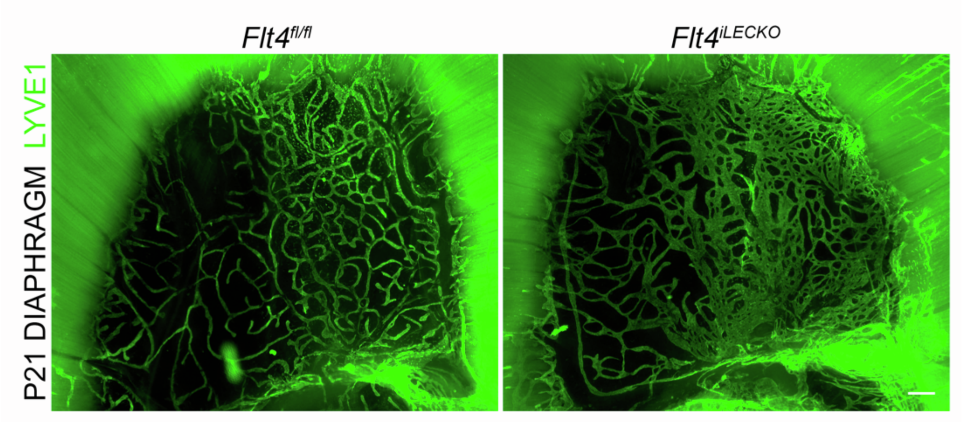


**Fig. S1. Lymphatic vessel hyperplasia in the diaphragm after postnatal deletion of *Flt4*.** To confirm presence of lymphatic capillaries in the P21 diaphragm, we stained for LYVE1 (green). LYVE1 positive lymphatic capillaries are present in both the $Flt4^{fl/fl}$ and the $Flt4^{iLECKO}$. N=3. Scale bar is 300 μm.

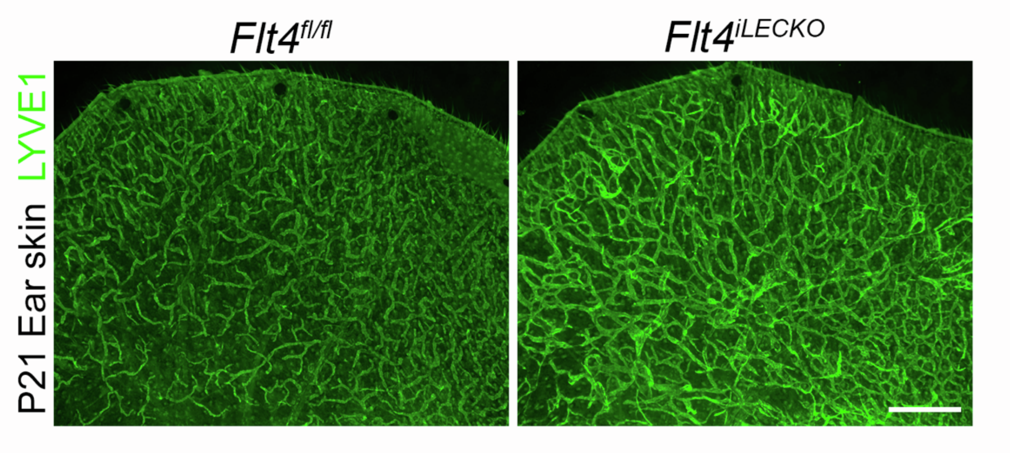


**Fig. S2. Lymphatic vessel hyperplasia in the ear skin after postnatal deletion of *Flt4*.** Lymphatic capillaries in the ear skin of both the *Flt4*$^{fl/fl}$ and the *Flt4*$^{iLECKO}$ mice stained positive for LYVE1 (green). N=3. Scale bar is 1 mm.

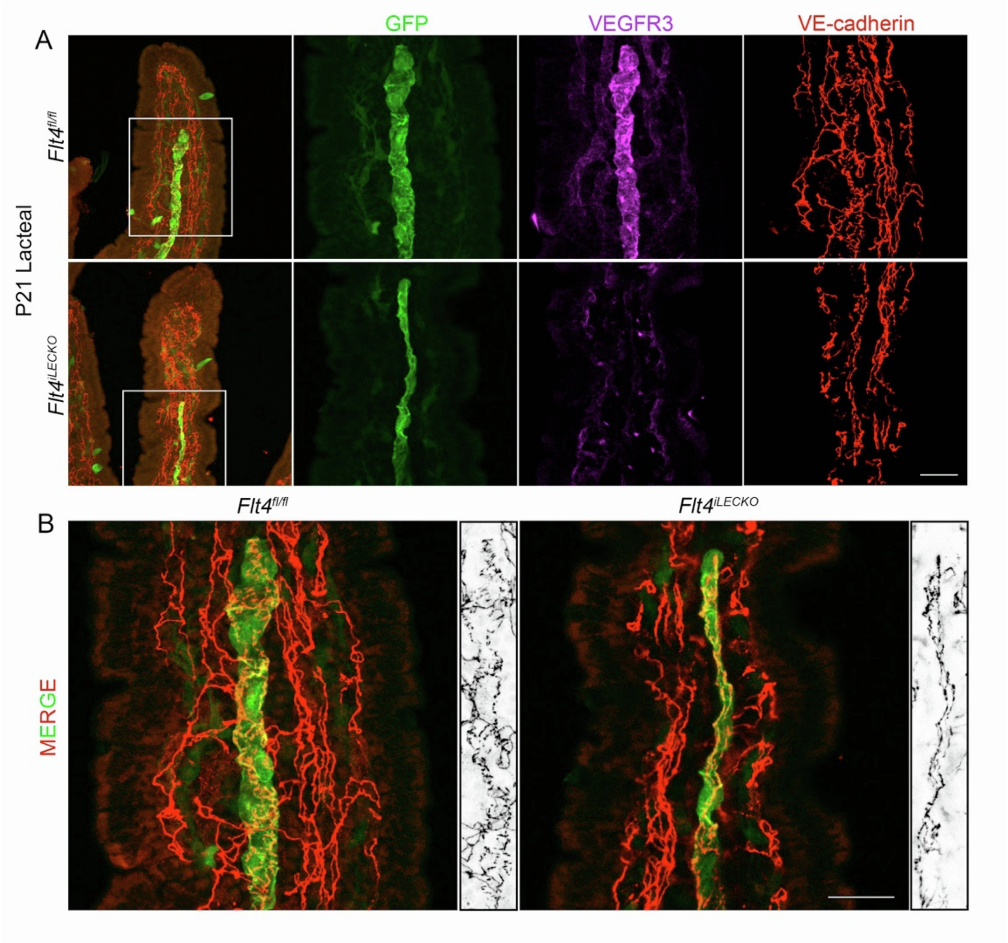


**Fig. S3. Impaired formation of button junctions in the intestinal lacteals of Flt4-iLECKO mice. (A)** Lacteals from P21 *Flt4*$^{fl/fl}$ controls and *Flt4*$^{iLECKO}$ mice were stained for GFP (green), VEGFR3 (magenta), and VE-cadherin (red). **(B)** Higher magnification images of the insets in **A**. VE-cadherin staining of the lacteal junctions is shown in grayscale. Scale bars are 25 µm.

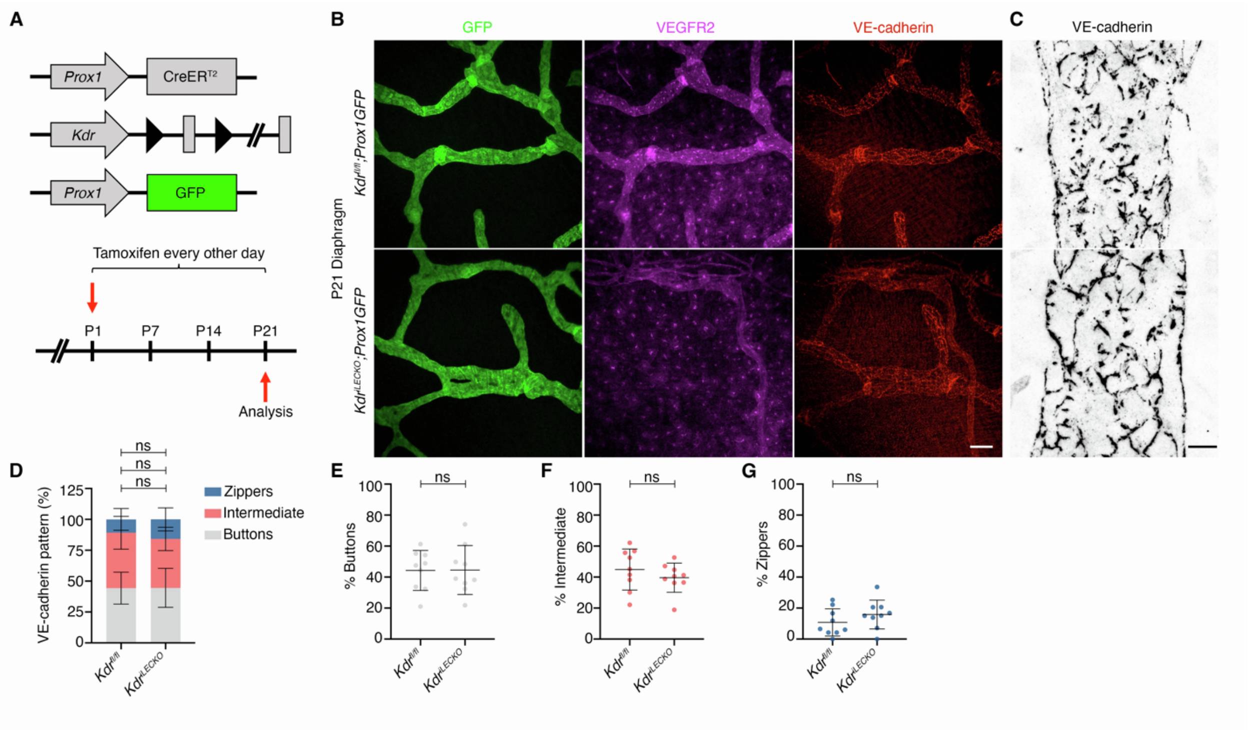


**Fig. S4. VEGFR2 is not required for button formation in the diaphragm.** (A) Tamoxifen schedule for deletion of *Kdr* to assess button formation. (B) P21 diaphragms were immunostained for GFP (green), VEGFR2 (magenta), and VE-cadherin (red). (C) Lymphatic vessel junction morphology at higher magnification. (D) Quantification of button, intermediate, and zipper junctions in *Kdr*$^{fl/fl}$ (N=3 mice; n=9 FOVs) and *Kdr*$^{iLECKO}$ mice (N=3 mice; n=9 FOVs). (E-G) Graphical breakdown of each junction type. Two-way ANOVA with Sidak's post hoc test was performed. ns = non-significant. Scale bar is 50 µm in B and 10 µm in C. FOVs, fields of view.

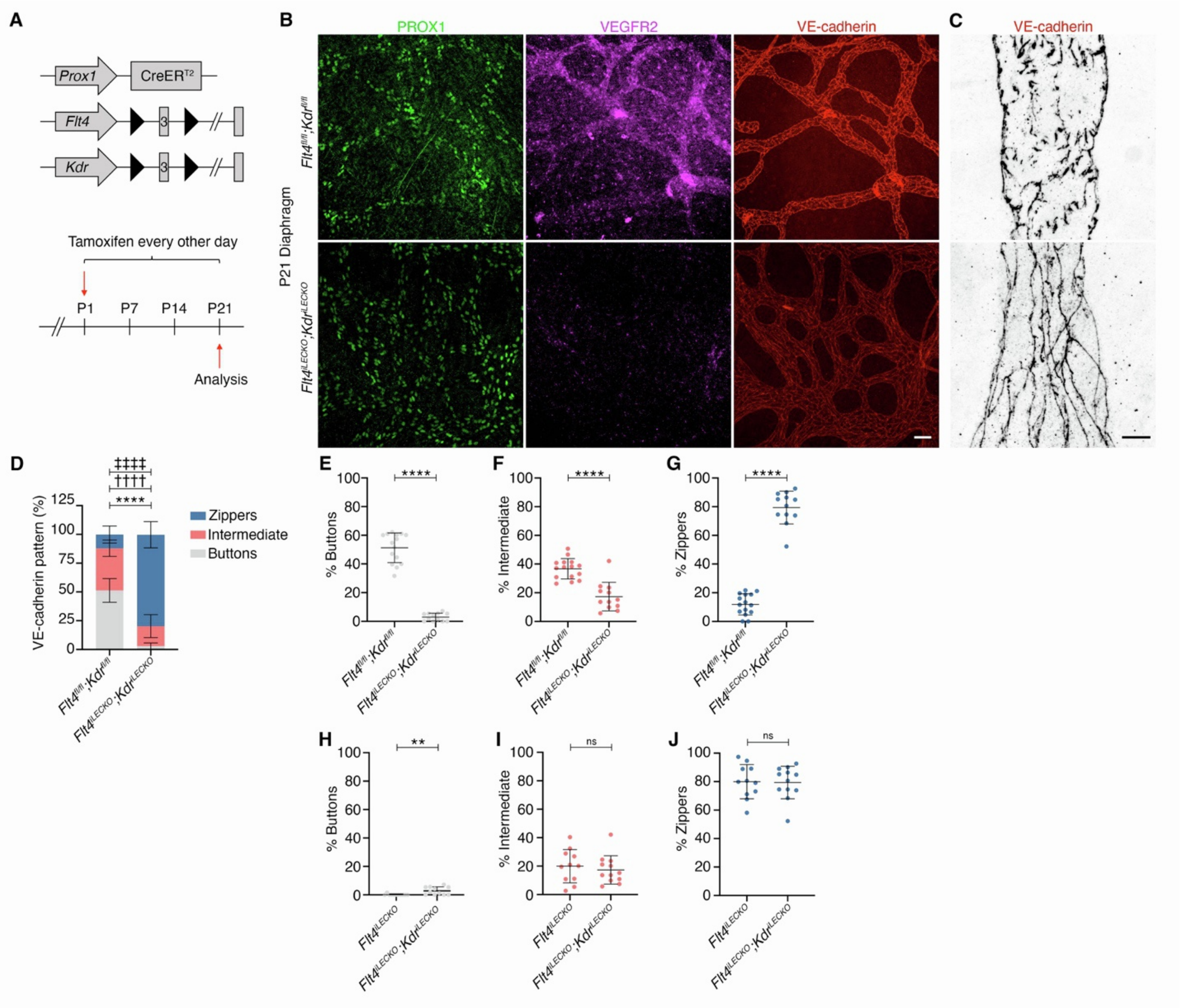


**Fig. S5. VEGFR2 signaling is not responsible for impaired button formation in VEGFR3 deficient lymphatic vessels. (A)** Tamoxifen schedule for simultaneous deletion of both *Flt4* and *Kdr* to determine if VEGFR2 is responsible for lack of button junctions in *Flt4*$^{iLECKO}$ mice. **(B)** Immunostaining of P21 diaphragms for PROX1 (green), VEGFR2 (magenta), and VE-cadherin (red). **(C)** Higher magnification of VE-cadherin immunostaining of intercellular junctions. **(D)** Quantification of button, intermediate and zipper junctions in *Flt4*$^{fl/fl}$*;Kdr*$^{fl/fl}$ (N=5 mice; n=15 FOVs) and *Flt4*$^{iLECKO}$*;Kdr*$^{iLECKO}$ (N=5 mice; n=12 FOVs). **(E-G)** Breakdown graphs of each junction type. **(H-J)** Comparison of *Flt4*$^{iLECKO}$ with *Flt4*$^{iLECKO}$*;Kdr*$^{iLECKO}$ double knockouts. Two-way ANOVA with Sidak's post hoc test. ****, $p$<0.0001 buttons; ††††, $p$<0.0001 intermediate; ‡‡‡‡, $p$<0.0001 zippers. Scale bar in B is 50 µm and in C, 10 µm. FOVs, fields of view.

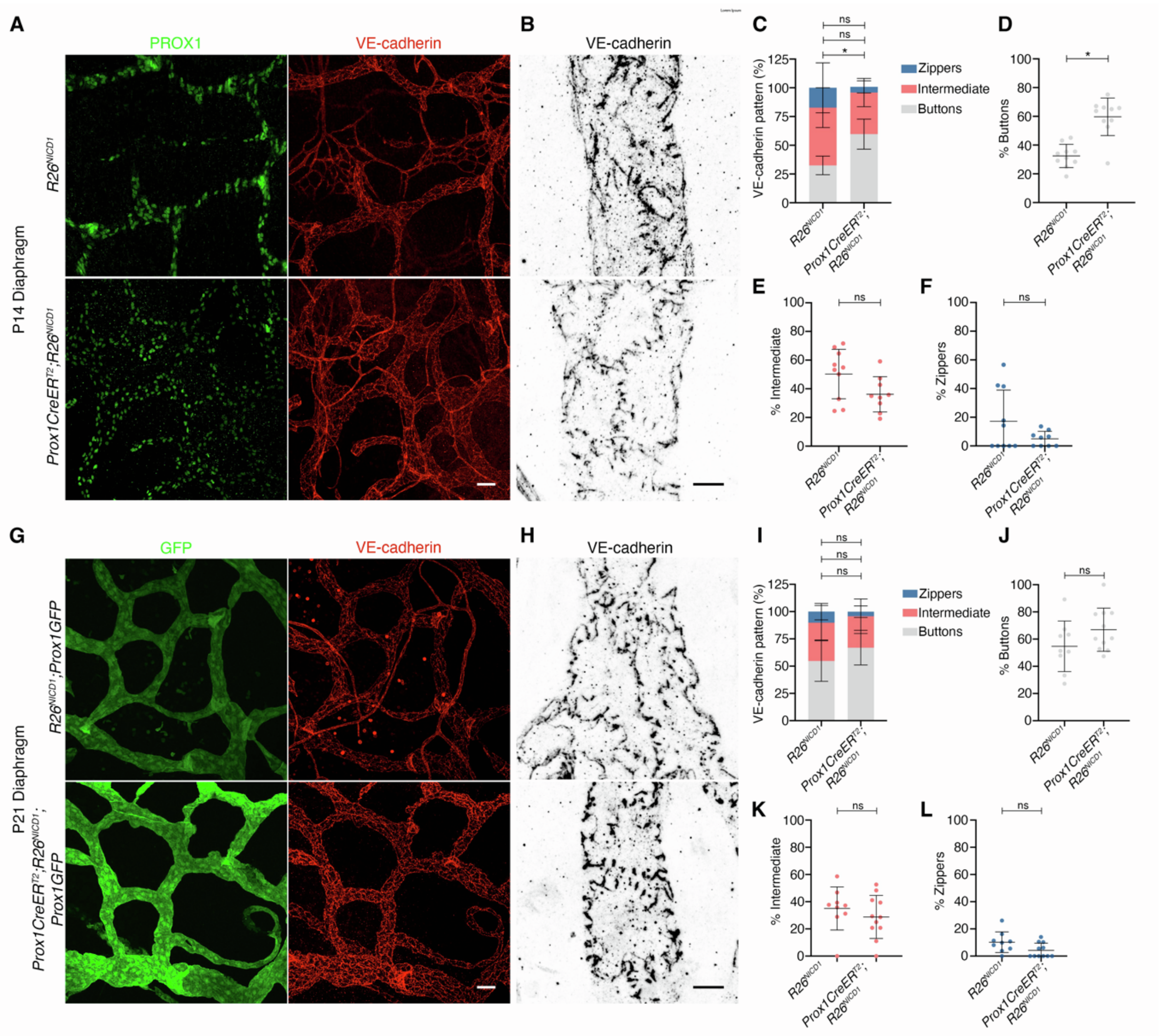


**Fig. S6. Overexpression of the NOTCH1 intracellular domain (NICD1) accelerates button formation in healthy mice.** **(A)** Constitutive NICD1 expression was induced with tamoxifen on P1 and P3, and lymphatic vessels in P14 diaphragms were immunostained for PROX1 (green) and VE-cadherin (red). **(B)** Higher magnification images of lymphatic vessel junction morphology. **(C)** Quantification of button, intermediate, and zipper junctions in $R26^{NICD1}$ controls (N=3 mice, n= 10 FOVs) and $Prox1CreER^{T2};R26^{NICD1}$ mice (N=3 mice; n= 10 FOVs). **(D-F)** Individual graphs of each junction type presented in **C**. **(G)** P21 diaphragms were immunostained for GFP (green) and VE-cadherin (red). **(H)** Higher magnification images to show lymphatic vessel junction morphology. **(I)** Quantification of junction types in $R26^{NICD1}$ controls (N=3 mice, n= 9 FOVs) and $Prox1CreER^{T2};R26^{NICD1}$ mice (N=3 mice; n=11 FOVs). **(J-L)** Graphical breakdown of button, intermediate and zipper junctions. Two-way ANOVA with Sidak's post hoc test. *, $p$<0.05 buttons; ns = non-significant. Scale bar in B and G is 50 µm and in C and H, 10 µm. FOVs, fields of view.
